# Diagnostic tools in childhood malaria

**DOI:** 10.1186/s13071-018-2617-y

**Published:** 2018-01-23

**Authors:** Amirah Amir, Fei-Wen Cheong, Jeremy R. De Silva, Yee-Ling Lau

**Affiliations:** Department of Parasitology, Faculty of Medicine, University Malaya, 50603 Kuala Lumpur, Malaysia

**Keywords:** Childhood, Malaria, Diagnosis, Microscopy, PCR, RDT

## Abstract

Every year, millions of people are burdened with malaria. An estimated 429,000 casualties were reported in 2015, with the majority made up of children under five years old. Early and accurate diagnosis of malaria is of paramount importance to ensure appropriate administration of treatment. This minimizes the risk of parasite resistance development, reduces drug wastage and unnecessary adverse reaction to antimalarial drugs. Malaria diagnostic tools have expanded beyond the conventional microscopic examination of Giemsa-stained blood films. Contemporary and innovative techniques have emerged, mainly the rapid diagnostic tests (RDT) and other molecular diagnostic methods such as PCR, qPCR and loop-mediated isothermal amplification (LAMP). Even microscopic diagnosis has gone through a paradigm shift with the development of new techniques such as the quantitative buffy coat (QBC) method and the Partec rapid malaria test. This review explores the different diagnostic tools available for childhood malaria, each with their characteristic strengths and limitations. These tools play an important role in making an accurate malaria diagnosis to ensure that the use of anti-malaria are rationalized and that presumptive diagnosis would only be a thing of the past.

## Background

First described in 2700 BC and recognized as one of the oldest known diseases on our planet, malaria is still a cause of devastation in many parts of the world [1]. Malaria is caused by a protozoan parasite of the genus *Plasmodium* [2]. The five *Plasmodium* species which cause malaria in humans are *P. falciparum*, *P. vivax*, *P. malariae*, *P. ovale* and *P. knowlesi* [3]. The former three species are distributed across Africa, Asia and South and Central America, whereas *P. ovale* is found in Africa and *P. knowlesi* in Asia [4]. Despite sharing the same geographical distribution, *P. vivax* infection is less frequently found in Africa compared to *P. falciparum*, but is the dominant species causing malaria in many regions outside Africa [4]. All five species are known to infect children [3, 5–7], with falciparum malaria being responsible for the majority of malaria-related deaths. Although the World Health Organization (WHO) has reported a fall in incidence and malaria deaths among populations at risk between the years 2010 and 2015, the estimated number of malaria deaths in 2015 remained high with a number close to 429,000, of which more than two thirds were in children under the age of five years old [8, 9].

Generally, children with malaria often present with fever, chills, headache, myalgia, vomiting and anorexia [3, 10]. Although severe malaria is often associated with *P. falciparum*, *P. vivax* monoinfection and mixed (*P. falciparum* and *P. vivax*) infection can also develop into severe malaria in children, as they too demonstrate respiratory distress, anemia and neurological manifestation [11]. While severe malaria has been reported in adults infected with *P. knowlesi* [12], little is known in children. Existing data showed that most children present with thrombocytopenia and anemia but did not show any progression into severe malaria [5, 13]. On the other hand, the least common *P. malariae* and *P. ovale* generally cause fever but the child does not appear ill [14].

Due to the non-specific manifestation of malaria infection, it is sometimes mistaken for gastroenteritis, pneumonia or sepsis [3, 15–17]. In contrast, since the symptoms of malaria are also exhibited by other microbial infection presenting with acute febrile illness, this may result in overestimation of malaria burden [18]. Diagnosis made based on clinical findings alone (presumptive diagnosis) may be unreliable and confirmatory laboratory diagnosis using diagnostic tools is crucial to ensure the accuracy of malaria diagnosis, thus, allowing administration of appropriate treatment [19]. Presumptive diagnosis has been shown to lead to over diagnosis and over treatment of malaria in children aged 1–5 years [20, 21]. Inappropriate use of antimalarial drugs in the past has led to the emergence of resistant malaria parasites [22, 23]. The newer ‘magic bullet’, artemisinin is not spared as *P. falciparum* in Myanmar, Cambodia, Thailand, Vietnam, Lao People’s Democratic Republic are seen to develop resistance [22, 24]. Furthermore, the latest WHO guidelines for the treatment of malaria has recommended artemisinin-based combination therapy (ACT) for adults and children [25]. Therefore, the WHO has come up with several recommendation under the Global Plan for Artemisinin Resistance Containment (GPARC) which include improving access to affordable and quality-assured malaria diagnostic tools to ensure that only confirmed cases receive treatment [26]. Suggestions have been made to change the policy from presumptive malaria treatment to laboratory-confirmed diagnosis and treatment [27–30]. In addition to being able to avoid unnecessary adverse reaction to antimalarial drugs, a laboratory-confirmed diagnosis and treatment also reduces drug wastage and minimizes the risk of parasite resistance development [31]. Over the years, malaria diagnostic tools have expanded, from the conventional microscopic examination of Giemsa-stained blood films to a myriad of serological and molecular methods which include the more commonly used rapid diagnostic tests (RDTs) and polymerase chain reaction (PCR) [19]. This review explores the different diagnostic tools currently available for childhood malaria with the aim of updating clinicians and researchers alike. With the array of diagnostic tools available, it is important that we work towards reducing presumptive diagnosis.

## Microscopic diagnosis

Accurate diagnosis with rapid and effective treatment is particularly important in children with malaria as most deaths occur within the first 24 h after admission to the hospital [32]. Direct microscopic observation of patients’ blood to detect malaria parasite remains the gold standard for malaria diagnosis [33, 34]. Microscopic examination of a stained thick blood film is done to determine the presence or absence of malaria parasite, whereas microscopic examination of a stained thin blood film allows *Plasmodium* species identification and parasitaemia quantification. Microscopic examination could also provide pathophysiological and prognostic information that can serve as indicators for the severity of disease, such as the morphological characteristics of the parasites, the maturity of asexual stages of the parasite and circulating pigment-containing phagocytes [35, 36]. The microscopic technique is widely used as it requires only a small volume of blood and it is cost effective compared to molecular techniques. Ngasala et al. [37] showed that microscopic diagnosis is useful in primary healthcare facilities as it helped to improve the appropriate management of non-malarial fevers and reduced the prescription of antimalarial drugs to children in Tanzania.

In ideal conditions, at least three negative serial blood smears (repeated every 12 h for 48 h) are needed before malaria diagnosis can be excluded [38, 39]. The detection limit of a well-trained microscopist can be as low as 5 parasites/μl blood, while average laboratory personnel may only report a positive blood smear at 50–100 parasites/μl of blood [14, 40]. The sensitivity and specificity of microscopic diagnosis varies greatly and are dependent on many factors. Ngasala et al. [37] showed that when 934 slides were examined by different microscopists, the overall sensitivity and specificity for detection of childhood malaria is 74.5% (95% CI: 69.8–78.7%) and 59.0% (95% CI: 54.9–62.9%), respectively, with a positive predictive value of 53.4% (95% CI: 49.0–58.0%) and a negative predictive value of 78.6% (95% CI: 74.0–82.0%). A study performed in Tanzanian children demonstrated that by comparing microscopy with RDTs and PCR, the sensitivity of conventional microscopy to detect pediatric malaria ranged from 26.3 to 100%, with the specificity ranged from 91.7 to 100% [41]. Discrepancy has also been found when microscopy and RDT yielded a positive result of 17 and 30%, respectively, in 515 Kenyan primary school children [42], whereas microscopy detected a 30% malaria prevalence in 230 Nigerian children compared to 24.3% using RDT [43]. Besides, the lack of malaria microscopist experts is another crucial factor in contributing to false reporting and errors in species identification. *Plasmodium knowlesi*, the fifth human *Plasmodium* could easily be misdiagnosed as *P. malariae* or *P. falciparum* under microscopy due to their morphological similarities [44]. Some children who were initially diagnosed with *P. malariae* infection via microscopy were confirmed to have *P. knowlesi* infection instead after PCR [5, 13]. These misdiagnoses could result in treatment delay and even fatal complications, as *P. knowlesi* can cause hyperparasitaemia within a short period of time and it causes a more severe disease compared to *P. malariae*. Sitali et al. [45] demonstrated that children under five years old have a higher frequency of acquiring mixed infection (infection with more than one *Plasmodium* species). However, mixed infections are often unrecognized or under-estimated by microscopists due to the tendency of one species dominating the other. This could lead to inadequate antimalarial drug treatment, particularly hypnozoites of *P. vivax* and *P. ovale*, the dormant form of parasites that can remain in the liver for many years, which can cause relapse. Difficulties in detecting parasites in low parasite density samples, overloaded laboratory personnel, ineffective quality control and assurance, poor condition of microscopes, and improper slide preparation can also lead to unreliable microscopy results [34, 46–48].

Besides the conventional bright field microscopic examination, additional techniques have been designed to improve malaria diagnosis via microscopy. The quantitative buffy coat (QBC) method is used to identify malaria parasites in peripheral blood by staining the DNA of parasites with acridine orange. QBC was found to be able to detect malaria in samples with low parasite numbers, as low as 5 parasites/μl blood [49]. Several studies proposed that QBC has a higher sensitivity compared to conventional microscopic examination in detecting malaria infection. Bosch et al. [50] demonstrated that QBC has a 100% sensitivity compared to microscopy examination in 37 indigenous children in Venezuela. Another study evaluated QBC on 720 schoolchildren and revealed a 99.6% sensitivity and 81.7% specificity on this technique, with 5.5% more sensitive than thick-film microscopic examination [51]. Similar results were obtained by Oloo et al. [52] where the overall sensitivity and specificity for QBC was 98 and 84% respectively, in a malaria survey performed on 360 Western Kenyan schoolchildren, with an accuracy of 92% and negative predictive value of 98%. Possible drawbacks of QBC are the difficulties in parasite species differentiation and subjective parasite quantification [53, 54]. Nonetheless, with its relatively reliable high sensitivity and specificity, QBC could still be useful as a supportive malaria diagnostic tool together with blood film microscopic screening in endemic field, as the processing of QBC is easier, faster, and requires less-trained personnel as compared to conventional microscopic examination [55–57]. For example in large-scale malaria screening, QBC could be performed prior to conventional bright-field microscopic examination to detect the presence of malaria parasites. Once malaria diagnosis has been established by the QBC, thin blood smears can then be used for accurate species identification and quantification of parasitaemia.

Another microscope-related technique, the Partec Rapid Malaria Test (PT) utilizes the Partec CyScope® (Partec GmbH, Münster, Germany), a microscope that has an extra incident UV light for detection of fluorescence light. The test slides used is readily labelled with an unspecific DNA binding fluorescent dye, 4′-6-diamidino-2- phenylindole (DAPI), that binds to intraerythrocytic *Plasmodium* DNA resulting in fluorescence. This method is easy and rapid, less labour intensive and requires less training time for laboratory personnel. Besides, the Partec CyScope® operates with integrated rechargeable batteries, which is convenient to be used in fields without electricity supply. Furthermore, it only requires few microliters of blood sample, which is ideal in paediatric patients. By using real-time PCR as gold standard, comparison study performed by Nkrumah et al. [58] in 489 children in Ghana reported the sensitivities of PT and microscopy examination were 62.2% (95% CI: 56.3–67.8%) and 61.8% (95% CI: 55.9–67.4%), respectively, for detection of malarial infections, with their specificities of 96.0% (95% CI: 92.3–98.3%) and 98.0% (95% CI: 95.0–99.5%), respectively. In a study of 541 Cameroonian schoolchildren using light microscopy as reference, PT was found to be 91.3% sensitive and 86.7% specific for detection of malaria [59]. Another study with 107 *Plasmodium* sp.-positive samples by microscopy demonstrated that both the sensitivity and specificity of PT for detection of childhood malaria [100% (95% CI: 96.6–100%) and 97.4% (95% CI: 93.6–99.3%), respectively] are higher than the sensitivity and specificity of RDT Binax Now® [97.2% (95% CI: 92.0–99.4%) and 93.6% (95% CI: 88.5–96.9%), respectively] [60]. Comparable sensitivity and specificity of PT to microscopic examination and RDT thus led to the proposal of using PT as an alternative malaria diagnostic tool in endemic areas. However, species differentiation could not be done using this test. Besides, the presence of non-specific artefacts, nuclei-containing cells such as reticulocytes, leukocytes and bacterial cells are very likely to lead to false positive results.

Conventional microscopy examination encounters many challenges, particularly the lack of trained malaria microscopists which could then lead to false diagnosis. To overcome this deficit, large efforts have been made in invention of computer-vision-based techniques and systems. These systems could function as “automated microscopists” and could greatly improve the speed, consistency and accuracy in malaria diagnosis. For instance, digital imaging scanning can be performed by SightDx first generation P1 and second generation P2 systems in which blood samples on test slides would be scanned automatically by the device with pre-set algorithms to detect the malaria parasites. These systems were able to scan the entire slide within few minutes, determine the parasitemia level and identify the *Plasmodium* species, with the performance result on par with human microscopist experts and many commercial RDTs [61, 62]. Recently, the latest commercial device Parasight platform was evaluated and demonstrates improved accuracy over the previous prototype P1 and P2 devices [63]. Despite above mentioned advantages, these computerized malaria diagnostic systems have yet to be evaluated on large scale.

## Rapid diagnostic tests

The RDT is an effective and important tool in malaria diagnosis and is increasingly seen as a complement to traditional diagnosis by microscopy. It forms the mainstay of diagnosis in resource-poor areas which do not have access to a laboratory or electricity and in these settings may supersede microscopy for diagnosis of malaria. According to the WHO, there are more than 200 malaria RDT products in the market [64] many of which have been assessed by independent studies according to WHO guidelines. According to the World Malaria Report 2015 [65], there were a total of 314 million RDT sales in 2014 and numbers are expected to increase over the years as the efficiency of these marketed RDTs increase.

Most RDTs work on the principle of capillary action. A capture and a separate detection antibody are used to provide a visual result where the capture antibodies are laid as a stripe on the membrane and the detection antibody is conjugated to an indicator, typically gold particles, that bind to the parasite antigen. This antigen-detection antibody complex binds to the capture antibody producing a visible line if the targeted antigen is present in the clinical sample [66]. Most RDTs are able to detect malaria antigens in 5–15 μl of blood and results can be obtained in 5–20 min depending on the manufacturer’s instructions. The detection limits for RDTs vary depending on individual manufacturers and the quality and the sensitivity of the RDTs depend on such factors as storage conditions, temperature and time of the assay [67].

There are currently three established antigens used for detection of *Plasmodium* in RDTs: *P. falciparum* histidine rich protein II (HRP-2), *Plasmodium* lactate dehydrogenase (pLDH) and aldolase. The HRP-2 protein is specific for *P. falciparum* detection while the pLDH and aldolase antigens are pan-malarial. Thus, most RDTs incorporate two of the three antigens to allow users to distinguish falciparum from non-falciparum infections. HRP-2 is a water soluble protein produced by asexual stages and young gametocytes of *P. falciparum* [68]. The pLDH enzyme on the other hand is produced by the sexual and asexual stages of *Plasmodium* and different isomers for this protein have been detected in various *Plasmodium* species [69]. Aldolase is an antigen utilized in the parasite glycolytic pathway and like pLDH is pan-malarial as well [70].

The HRP-2 antigen has been shown to have better sensitivity compared to pLDH although specificity was found to be better with pLDH [71]. Furthermore, HRP-2 is less expensive, has a lower detection threshold and is stable at a wider range of temperatures thus making it the more widely used antigen [72]. The drawbacks of this antigen however, are that it only is able to detect *P. falciparum* infection and any antigenic variation may give a false-negative result [73]. Furthermore, HRP-2 antigens are known to still be in the circulation of the patient weeks after clearance of the parasite [74] thus making it unsuitable to be used in assays for monitoring response to drug treatment.

More recently, there have been reports of deletions of the HRP-2 in several areas endemic for *P. falciparum* infection. The prevalence of parasites with the HRP-2 gene deletions may, however, vary in differing localities. HRP-2 deletions leading to false negative reports have been published in Mali [75], Rwanda [76], Colombia [77], Ghana [78], Kenya [79], the Democratic Republic of Congo [80] and India [81]. Guidelines issued by the WHO, however, indicate that deletion of the HRP-2 gene is not likely to be the main cause of false-negative results in RDTs and point towards other more probable causes including poor transport and storage conditions, operator errors, or the use of poor quality RDTs or the use of a wrong comparator such as poor-quality microscopy for cross-referencing of negative diagnostic results [82]. The guidelines do state that HRP-2 deletion should be suspected in a specific number of instances and PCR may be used to confirm the diagnosis and the deletion of the HRP-2 gene.

The use of RDTs for malaria diagnosis in children suffer the same limitations that affect the use of the diagnostic method in adults. The primary limitations of this test include reduced sensitivity for non-falciparum malaria, and a lack of accuracy in extreme environmental conditions such as those found in field situations where it ironically is used the most [83, 84]. However, the RDT’s ease of use and interpretation and its ability to be deployed in the field makes it an invaluable tool for diagnosis of malaria in children in rural areas. A study by Smart et al. [85] on pediatric inpatients in a two Tanzanian referral hospitals found moderate agreement between the use of microscopy and RDT for diagnosis of patients but suggested that RDT are a better initial test in diagnosing malaria among pediatric patients. The author also argues that the use of RDT would reduce the rate of overtreatment in hospitals in Tanzania which indirectly would provide a significant cost saving. Furthermore, withholding antimalarial drugs from children who test negative in an RDT test is a safe practice in an outpatient setting [86]. The use of RDT in hospitals would also decrease turn-around time providing physicians with feedback and results in a timely manner. Overtreatment of children was also reported in Samara hospital, Nigeria where 57% of febrile children admitted to the hospital received antimalarial medication for treatment of presumed malaria before the presentation [87]. When evaluating the sensitivity and specificity of RDT for diagnosis of pediatric malaria, the authors found that RDT had a sensitivity of 40.3% and a specificity of 89.6%. However, it was of note that the sensitivity of the test dropped drastically with lower parasite densities. These results were contradictory to a previous study on the effectiveness of RDT in febrile children in Sokoto, Nigeria, where sensitivity of RDT was found to be 93% [88].

Recent advancements in RDTs for malaria diagnosis include the development of the ultra sensitive rapid diagnostic test (uRDT) which has higher sensitivity compared to the conventional RDTs. A recent study by Das et al. [89] on the Alere™ Malaria Ag P.f uRDT showed a greater than 10-fold lower detection limit for the HRP-2 antigen compared to a conventional RDT in both a high and low transmission setting. The study also indicates high specificitiy for this uRDT relative to quantitative PCR (qPCR) and histidine-rich protein II (HRP2) enzyme linked immunosorbent assay (ELISA). Furthermore, the uRDT was able to detect new infections 1.5 days sooner indicating overall improved diagnostic performance characteristics when compared to conventional RDTs [89].

Overall, studies on the sensitivity and specificity of RDT in diagnosing malaria have been positive in both a laboratory and outpatient clinical setting [90–92] and thus should be considered a useful tool for pediatric malaria diagnosis.

## Molecular diagnostic methods

Molecular techniques, such as PCR, have gained much attention and significance in malarial diagnosis, especially after the discovery of knowlesi malaria in humans [93, 94]. This method enables the specific identification of malarial parasites up to the species level. Furthermore, it is highly sensitive when compared to microscopy. The theoretical detection limit of PCR can be as low as 0.02 parasites/μl [95, 96] with nested PCR being the most sensitive nucleic acid amplification technology thus far [41, 97] versus an experienced microscopist, which is said to have a detection limit of approximately 5 parasites/μl [40]. With other diagnostic methods falling short in terms of practicality, cross-reactivity problems, or having incomplete coverage of all medically important Plasmodia [98, 99], PCR-based methods seem promising as the new gold standard in malarial diagnosis, especially in cases with low parasitemia or in the case of mixed species [100].

The molecular amplification of the small subunit, 18S, of ribosomal RNA (18SrRNA) was first carried out by Snounou et al. [101] using a nested PCR technique, the most widely-used PCR method in malarial diagnostic research. The sensitivity of this molecular method was found to be greater than RDTs and microscopy in a study conducted by Mens et al. [102]. In their work, 338 children with the clinical symptoms of *Plasmodium* infection in Tanzania and Kenya were analyzed with microscopy, RDT, or molecular method. Molecular testing found a substantially higher amount of positive samples compared with RDTs and microscopy, confirming the elevated sensitivity of PCR [103] which enabled the identification of more children with low parasitemia. Around 40–42% the samples collected in Kenya were found positive with molecular methods, with parasite counts ranging from 16 to 108 parasites/ml blood. Similarly in Tanzania, 13–14 samples were found positive with molecular methods with parasite counts ranging from 9 to 170,000 parasite/ml blood. Additionally, molecular methods have the potential to detect malarial parasites in asymptomatic infections. These undetected sub-microscopic infections, though less common in children than in adults, may still be able to infect mosquito vectors and could reintroduce malaria into certain regions. Semi-nested PCR based on the 18S small subunit ribosomal RNA (ssrRNA) gene permitted the identification of a high number of children (80%) infected with *P. falciparum*. These children had all initially tested negative with the microscopy method [104]. Interestingly, others have found the high copy number of cytochrome *b* PCR to be more sensitive than *18S* rDNA PCR [41, 105, 106]. Hsiang et al. [107] detected three times as many infections with cytochrome *b* nested PCR than by microscopy (15/472 *vs* 4/472) among asymptomatic children with lowest detection limit of 10 parasites/μl. This technique also performed better than single-round PCR and real-time methods [107]. A more recent study by Mahende et al. [108] determined 21 malaria RDT-positive samples from Tanzanian children while microscopy was negative; six samples were detected positive by *18S* rDNA PCR [108]. Of note is that eight samples that were RDT-negative but microscopy-positive were confirmed to possess *P. falciparum* species through PCR. Three-quarters of the 867 malaria patients from this study had low levels of parasitaemia. Therefore, PCR is a worthy alternative as the interpretation of the results either by agarose gel observation or Ct value does not require specialized skill and is not altered by the subjectivity of the observer [95]; this is the precise opposite to microscopy where specific training is required for species differentiation.

The specificity of PCR has also been demonstrated by Nsobya et al. [109]. Upon enrollment, 55 (17%) of 316 asymptomatic children in Uganda were found to be infected with *P. falciparum* via microscopy (parasite densities was 16–71,840 parasites/ml). By using species-specific nested PCR, the prevalence of malaria was observed to be 148 (47%), of which 36% were *P. falciparum*, 18% *P. falciparum* mixed infection, 10% *P. ovale*, 7% *P. vivax*, 4% *P. malariae*, and 3% non-*P. falciparum* mixed infection. Two children were PCR negative but microscopy positive [109].

Real-time quantitative PCR (qPCR) and nucleic acid sequence-based amplification (QT-NASBA) assays can also be utilized to determine parasite density. The advantage of qPCR over other molecular techniques is the quantification of parasitic densities. By correlating quantification by two commercial qPCR (PrimerDesign Ltd., Alicante, Spain) kits, Santana-Morales et al. [109] confirmed that qPCR is an accurate means to quantify parasitic densities. One case of imported falciparum malaria was reported in France in a boy that visited his family in Africa during a summer break. qPCR quantified *P. falciparum* DNA levels in an effort to monitor the parasitemia under treatment and to determine chloroquine resistance. qPCR was able to detect and quantify infections that have very low infection (0.001%) [110]. qPCR assays targeting the high-copy telomere-associated repetitive element 2 (TARE-2) and the var. gene acidic terminal sequence (varATS) were also developed for ultra-sensitive detection of *P. falciparum*. Compared to TARE-2 or varATS qPCR, *18S* rRNA gene qPCR was unable to identify 48 infections in 498 samples from a malaria survey undertaken in Tanzania [111].

In a recent report, a reverse transcription-polymerase chain reaction (qRT-PCR) was developed for the asexual *18S* rRNAs of *P. falciparum* and *P. vivax*. qRT-PCR demonstrated high sensitivity as compared to qPCR by detecting 34/52 symptomatic patients and 13/36 asymptomatic patients [112]. Other molecular techniques developed for malaria diagnosis include loop-mediated isothermal amplification (LAMP), flow cytometry, and microarray [113–115]. However, the efficiency of these methods has not been established for childhood malaria.

A lack of clear consensus on standardized methods for PCR makes it difficult to interpret and compare results. There is a need to develop guidance on indications for use, assay selection, and quality assurance/control for PCR and other molecular diagnostic techniques for the specific conditions in which employing malaria diagnostic tools may be appropriate. To ensure the data obtained from PCR-based method are reliable, users are strongly encouraged to follow the WHO external quality assurance scheme for malaria nucleic acid amplification testing [116]. Another drawback is that PCR is cumbersome, expensive, and requires well-trained staff with stringent laboratory cleanliness. Such criteria may not be fulfilled in certain laboratory settings, especially those in remote areas in developing countries [117].

## Non-invasive tests

The future of malaria diagnostic tools is exciting as we start to see the development of new non-invasive methods. One such method is by detecting specific thioeter levels in human breath which acts as biomarkers. Exhaled breath is analyzed using gas chromatography-mass spectrometry. An early clinical trial of this method on adult volunteers infected with *P. falciparum* showed promising results with thioether levels appearing to have the same periodicity as the parasite’s 48 h erythrocytic life-cycle [118].

The urine malaria test (UMT) kit, a recombinant monoclonal antibody based test was also recently developed to detect *P. falciparum* specific HRP-2, a poly-histidine protein or fragments present in the urine of febrile patients. Similar to most RDTs, UMT works on the principle of capillary action. This method involves dipping the UMT strip into 200 μl urine for 2 min and results read after 20 min based on the lines appearing on the strip. Initial assessment on patients in South-East Nigeria showed that it was comparable with the microscopy technique [119]. Another multicenter UMT trial in Lagos state, Nigeria, managed to include patients infected with *P. falciparum*, *P. vivax*, *P. malaria* and *P. ovale* [120]. Here, they found that UMT sensitivity and specificity was 93 and 83%, respectively, when used among febrile children under the age of five. The performance of the UMT in this study was found to be comparable with that of BinaxNow, a blood-based malaria RDT.

Several studies involving a mixed population consisting of adults and children have tried using urine and saliva as alternative DNA sources for malaria diagnosis by PCR with promising results [121, 122]. Furthermore, saliva has also been used for the quantitative detection of *P. falciparum* HRP-2 antigen using ELISA with encouraging outcome [123, 124].

Another fascinating method recently developed was the transdermal detection of vapor nanobubbles around intraparasite hemozoin using a prototype device [125]. This method involves delivering laser pulses to blood through the skin using a probe after which, the responding acoustic traces are collected simultaneously with laser irradiation and analyzed.

These non-invasive methods serve as a potential alternative tool in malaria diagnosis especially where there are difficulties in obtaining blood samples (particularly in children), problems with carrying out conventional diagnostic method or when safety concerns are expressed. However, most of these non-invasive tests are still at their infancy and there is a need for optimization of the technology and further testing to also include field-acquired infection and paediatric populations.

## Conclusions

The characteristic features of microscopic diagnosis, rapid diagnostic test and molecular diagnostic methods are summarized in Table 1. Despite the progress and advancement of various malaria diagnostic tools, the microscopy technique remains the gold standard for malaria diagnosis. RDTs prove to be valuable as it can be undertaken without any basic laboratory infrastructure or trained personnel. The ability of RDTs to produce results within a few minutes help reduce over-treatment or misdiagnosis from presumptive diagnosis. Nevertheless, because of the variable sensitivity and specificity of different RDTs, WHO procurement guidelines should be followed when procuring malaria RDTs and a good quality control/assurance programme should be implemented. The highly sensitive and specific results obtained from PCR makes it a good candidate as the next gold standard for malaria diagnosis. However, due to it being costly, requiring trained personnel and a proper laboratory setting, this method is not practical especially in rural or out-patient settings. Light microscopy and RDTs are still the mainstay of malaria diagnosis in basic healthcare systems, whereas molecular methods may be utilized in advanced healthcare systems as part of malaria diagnostic protocol. In elimination settings, molecular methods are required to complement field diagnostics (microscopy and RDTs) in order to identify asymptomatic carriers which is critical to the success of elimination programmes [126].

**Table 1:** Summary of the three main diagnostic methods in childhood malaria with their characteristic features

Method		Key characteristics	Parasite species detectable	Advantages	Disadvantages	Reference
Microscopic diagnosis	Conventional bright field microscopic examination	Giemsa-stained thick blood film to determine the presence or absence of malaria parasite	*Plasmodium* genus-specific	- Small amount of sample (blood) is required; - Able to quantify parasitaemia; - Provides prognostic information that serves as indicator for disease severity (morphological characteristics of the parasites, the maturity of asexual stages of the parasite); - Cost effective compared to molecular techniques	- Difficulties in detecting parasites in low parasite density samples (50–100 parasites/μl); - Malaria microscopist expert/well-trained personnel is needed to interpret the result (high morphological similarities between *P. falciparum*, *P. malariae* and *P. knowlesi* could lead to misdiagnosis and treatment delay)	[5, 13, 14, 33–48]
		Giemsa-stained thin blood film to identify the *Plasmodium* species	All human *Plasmodium* species			
	Quantitative buffy coat method (QBC)	Detection of malaria parasites in centrifuged peripheral blood by staining the parasite DNA with acridine orange and examination under fluorescence microscope	*Plasmodium* genus-specific	- Higher sensitivity (5 parasites/μl) compared to bright field microscopic examination; - Fast and easy to be performed; - Interpretation of the result is simple and requires less-trained personnel	- Difficulties in parasite species differentiation and subjective parasite quantification; - Specific equipment (fluorescence microscope) is required	[49–57]
	Partec Rapid Malaria Test (PT)	Detection of malaria parasites using test slide that is readily labelled with 4′-6-diamidino-2- phenylindole (DAPI) which binds to intraerythrocytic *Plasmodium* DNA, resulting in fluorescence under Partec CyScope® (fluorescence microscope)	*Plasmodium* genus-specific	- Easy and rapid, less labour-intensive and requires less training time for laboratory personnel; - Could be used in the field without electricity supply; - Small amount of sample (few μl) is required	- Difficulties in species differentiation; - False positive results due to the presence of non-specific artefacts or nuclei-containing cells (reticulocytes, leukocytes and bacterial cells); -Specific equipment (Partec CyScope®) is required	[58–60]
Rapid diagnostic test (RDT)	OptiMAL®	Detection of malaria via the pLDH antigen	*P. falciparum* and *P. vivax*	Ease of use, rapid diagnosis and result interpretation, sensitive, field-deployable	Less sensitive compared to molecular diagnostic methods, heat sensitive, reduced sensitivity for non-falciparum malaria, false-negative results due to low-level expression or deletion of target antigen genes (*pfhrp2*)	[83]
	ParaSight-F test	Detection of malaria via the HRP-2 antigen	*P.falciparum*			[84]
	Immunochromatographic test (ICT) Malaria PF test	Detection of malaria via the HRP-2 antigen	*P.falciparum*			[84]
	SD Bioline Malaria AG Pf/Pan	Detection of malaria via the HRP-2 and pLDH antigen	*P. falciparum* (HRP-2), pan-malarial (pLDH)			[85]
	CareStart™ Malaria	Detection of malaria via the HRP-2 and pLDH antigen	*P. falciparum* (HRP-2), pan-malarial (pLDH)	High specificity and PPV	Low sensitivity at low parasite densities	[87]
	Malaria pf Rapid device	Detection of malaria via the HRP-2 antigen	*P. falciparum*	Sensitivity and specificity comparable to those for light microscopy		[88]
	Ultra sensitive RDT (uRDT)	Detects HRP-2 antigen of *P. falciparum* malaria	*P. falciparum*	Higher sensitivity, specificity and ability to detect new infections faster than conventional RDT	Similar to conventional RDTs, is less sensitive compared to molecular diagnostic methods	[89]
Molecular diagnostic methods	Nested PCR	Targeting *18S* rRNA gene	*Plasmodium* genus-specific	Elevated sensitivity compared to RDTs and microscopy	Cumbersome, expensive, and requires well-trained staff with stringent laboratory cleanliness to minimize risk of contamination	[101]
		Targeting *18S* rRNA gene	*Plasmodium* genus-specific followed by nested species-specific PCR	More sensitive than microscopic examination for identification of asymptomatic malaria		[108]
		Targeting *cytochrome b* gene	*Plasmodium* genus-specific	Detection limit of 10 parasites/μl, better than single-round PCR and real-time methods		[41, 104, 105, 107]
	Semi-nested PCR	Targeting *18S* rRNA gene	*P. falciparum* and *P. vivax*	More sensitive than microscopic examination for identification of sub-microscopic infections		[103]
	Quantitative nucleic acid sequence-based amplification (QT-NASBA)	Targeting *18S* rRNA gene. Quantification was achieved by co-amplification of the RNA in the sample with one modified in vitro RNA as a competitor	*P. falciparum*	- Fast, sensitive, reliable, and quantitative; - Allowed for the sub-microscopic quantification; - Detection limit of 10 parasites/μl		[101, 102]
	Multiplex PCR	Targeting *18S* rRNA gene	*P. falciparum* and *P. vivax*	- Detection limit of 0.1 parasites/μl; - No cross-reaction between *Plasmodium* spp.; - Able to detect the mixed infection		[106]
	Real-time quantitative PCR (qPCR)	Targeting *plasmepsin 4* in *P. falciparum* and the *aspartic protease PM4* in *P. vivax*	*P. falciparum* and *P. vivax*	- Quantification of parasite densities; - More sensitive than microscopic examination; - Detection limit of 5.6 copies/μl; - Able to detect and quantify infections that have very low infection (0.001%)		[103]
	qPCR	Targeting *telomere-associated repetitive element 2* and the *var.* acidic terminal sequence	*P. falciparum*	- Detection limit of 0.03 to 0.15 parasites/μl; - 10× more sensitive than standard *18S* rRNA qPCR		[110]
	Reverse transcription-polymerase chain reaction (qRT-PCR)	Targeting *18S* rRNA	*P. falciparum* and *P. vivax*	Able to detect and differentiate submicroscopic malaria infections as low as 10 parasites/ml and 18 copies/μl		[111]

## Abbreviations

LAMP: Loop-mediated isothermal amplification
PCR: Polymerase chain reaction
QBC: Quantitative buffy coat
qPCR: Real-time quantitative polymerase chain reaction
RDT: Rapid diagnostic tests
